# Region‐specific differences and areal interactions underlying transitions in epileptiform activity

**DOI:** 10.1113/JP277267

**Published:** 2019-02-13

**Authors:** Neela K. Codadu, R. Ryley Parrish, Andrew J. Trevelyan

**Affiliations:** ^1^ Institute of Neuroscience Medical School Framlington Place Newcastle upon Tyne NE2 4HH UK; ^2^ Department of Neurology Columbia University New York NY 10032 USA

**Keywords:** epilepsy, hippocampus, interictal discharges, neocortex, seizures

## Abstract

**Key points:**

- Local neocortical and hippocampal territories show different and sterotypical patterns of acutely evolving, epileptiform activity.
- Neocortical and entorhinal networks show tonic–clonic‐like events, but the main hippocampal territories do not, unless it is relayed from the other areas.
- Transitions in the pattern of locally recorded epileptiform activity can be indicative of a shift in the source of pathological activity, and may spread through both synaptic and non‐synaptic means.
- Hippocampal epileptiform activity is promoted by 4‐aminopyridine and inhibited by GABA_B_ receptor agonists, and appears far more sensitive to these drugs than neocortical activity.
- These signature features of local epileptiform activity can provide useful insight into the primary source of ictal activity, aiding both experimental and clinical investigation.

**Abstract:**

Understanding the nature of epileptic state transitions remains a major goal for epilepsy research. Simple *in vitro* models offer unique experimental opportunities that we exploit to show that such transitions can arise from shifts in the ictal source of the activity. These transitions reflect the fact that cortical territories differ both in the type of epileptiform activity they can sustain and in their susceptibility to drug manipulation. In the zero‐Mg^2+^ model, the earliest epileptiform activity is restricted to neocortical and entorhinal networks. Hippocampal bursting only starts much later, and triggers a marked transition in neo‐/entorhinal cortical activity. Thereafter, the hippocampal activity acts as a pacemaker, entraining the other territories to their discharge pattern. This entrainment persists following transection of the major axonal pathways between hippocampus and cortex, indicating that it can be mediated through a non‐synaptic route. Neuronal discharges are associated with large rises in extracellular [K^+^], but we show that these are very localized, and therefore are not the means of entraining distant cortical areas. We conclude instead that the entrainment occurs through weak field effects distant from the pacemaker, but which are highly effective at recruiting other brain territories that are already hyperexcitable. The hippocampal epileptiform activity appears unusually susceptible to drugs that impact on K^+^ conductances. These findings demonstrate that the local circuitry gives rise to stereotypical epileptic activity patterns, but these are also influenced by both synaptic and non‐synaptic long‐range effects. Our results have important implications for our understanding of epileptic propagation and anti‐epileptic drug action.

## Key points

- Local neocortical and hippocampal territories show different and sterotypical patterns of acutely evolving, epileptiform activity.
- Neocortical and entorhinal networks show tonic–clonic‐like events, but the main hippocampal territories do not, unless it is relayed from the other areas.
- Transitions in the pattern of locally recorded epileptiform activity can be indicative of a shift in the source of pathological activity, and may spread through both synaptic and non‐synaptic means.
- Hippocampal epileptiform activity is promoted by 4‐aminopyridine and inhibited by GABA_B_ receptor agonists, and appears far more sensitive to these drugs than neocortical activity.
- These signature features of local epileptiform activity can provide useful insight into the primary source of ictal activity, aiding both experimental and clinical investigation.

## Introduction

Epilepsy is a condition that is defined by sudden transitions from a functional brain state into pathological states. These transitions are associated with dramatic changes also in the electrophysiological signals, and indeed EEG recordings provide a very sensitive assay of brain states. The interpretation of these signals, though, is often difficult, and in most cases we still do not understand what biological processes underlie the key shifts in the electrophysiological signal. They do, though, offer great potential for providing advance warning about imminent seizures, and so warrant further study.

Epileptic transitions can arise from local network interactions (Bernard *et al*. 2000; Ziburkus *et al*. 2006; Huberfeld *et al*. 2011; Trevelyan & Schevon, 2013; Avoli *et al*. 2016) or cellular changes, such as intracellular chloride concentration (Dzhala *et al*. 2010; Pavlov *et al*. 2013; Ellender *et al*. 2014; Pallud *et al*. 2014). A role in these transitions has also been hypothesized for larger scale network interactions (Kramer *et al*. 2012; Martinet *et al*. 2017; Liou *et al*. 2018). To address this hypothesis, it is extremely helpful to identify where the source of the pathological discharges is, whether there can be more than one source, and if so, which is primary, and how they might switch; this detail can then further provide insight into how epileptic activity spreads. Most epileptic seizures are thought to arise from pathology located in hippocampal, parahippocampal or neocortical circuits, but it remains unclear to what extent the pathological activity is set by the intrinsic excitability of the local networks (Traub & Wong, 1982; Miles & Wong, 1983; Prince & Connors, 1984; Dichter & Ayala, 1987; Ziburkus *et al*. 2006) or interactions between the areas (Miles *et al*. 1984; McCormick & Contreras, 2001). Brain slice preparations offer unique experimental opportunities for recording, manipulating and isolating network activity. These preparations have yielded many insights into a wide range of topics from cellular excitability and synaptic interactions up to network dynamics, for instance by providing a framework to understand human recordings (Schevon *et al*. 2012; Smith *et al*. 2016) where the potential for invasive investigation is greatly limited.

We set out to investigate the role of interactions between brain areas in epileptic transitions. An important series of studies using the 0 Mg^2+^ model (Swartzwelder *et al*. 1986b; Mody *et al*. 1987; Anderson *et al*. 1990; Dreier & Heinemann, 1990, 1991; Bragdon *et al*. 1992; Morrisett *et al*. 1993; Zhang *et al*. 1995; Dreier *et al*. 1998) characterized a notable transition from an early tonic–clonic patterns of epileptiform discharges into a different, recurrent pattern of discharge. The nature of this critical transition, however, has remained a mystery. Of added interest is that this transition is associated with a marked change in the pharmaco‐sensitivity of the pathological discharges (Heinemann *et al*. 1994b). Since the various brain areas may differ in how epileptic discharges are manifest, we hypothesized that a key component of this transition might reflect changes in the level of involvement of different brain territories.

These prior studies all used brain slices prepared from adult rats, but have not been repeated using tissue from other species. We now show that the same evolution of activity is also seen in mouse brain slices, thereby opening up this phenomenon for further study in transgenic animals carrying mutations relevant for human epilepsy. We then identify an important correlate of the transition, which is the surprisingly late involvement of hippocampal activation in this model, and which subsequently acts as a pacemaker, entraining activity in other cortical networks. Interestingly, the entrainment of overlying neocortex does not require intact synaptic pathways, but instead can arise from field effects secondary to focal discharges (Jefferys & Haas, 1982; Jefferys, 1995; Frohlich & McCormick, 2010; Anastassiou *et al*. 2011). We further show that the entrainment does not happen through the diffusion of extruded K^+^, because the rise in extracellular [K^+^] associated with epileptiform discharges is very focal. Finally, the site of the dominant epileptiform activity in these preparations is highly sensitive to drugs that affect K^+^ conductance. The GABA_B_ agonist is a very powerful suppressor of the hippocampal focus, and shifts the slice back towards the neo‐/entorhinal cortical pattern of tonic–clonic‐like discharges, whereas the K^+^ channel blocker 4‐aminopyridine strongly promotes hippocampal activity, far more rapidly than in the other areas, the exact opposite of the evolving pattern induced by 0 Mg^2+^. These results show that locally recorded transitions in the pattern of epileptiform discharge may arise from the new involvement of distally located epileptic circuits. These changes thus reflect which cortical territories are involved and how the activity spreads to other networks. These models illuminate a variety of epileptic phenomena, including the evolution of epileptic foci, sudden shifts from one focus to another, and how different cortical areas show distinctive patterns of epileptic discharge and propagation. As such, they can provide a wealth of metrics for comparing anti‐epileptic drugs, and for understanding phenotypes in genetic models of epilepsy.

## Methods

### Ethical approval

All animal handling and experimentation were done according to the guidelines laid by the UK Home Office and Animals (Scientific Procedures) Act 1986, and were approved by the Newcastle University Animal Welfare and Ethical Review Body (AWERB reference no.: 545). All mice used in this study were housed in individually ventilated cages in a 12 h light, 12 h dark lighting regime. All mice were provided with food and water *ad libitum*.

### Slice preparation

Young adult, male and female mice (wild‐type C57‐Bl6 strain from The Jackson Laboratory (stock no. 000664; Bar Harbor, ME, USA), age 2–3 months) were sacrificed by a ‘Schedule 1’ method of cervical dislocation. The brains were removed and sliced horizontally (400 μm thickness) in ice‐cold artificial cerebrospinal fluid (ACSF; containing (in mm): 3 MgCl_2_, 126 NaCl, 26 NaHCO_3_, 3.5 KCl, 1.26 NaH_2_PO_4_, 10 glucose), using a Leica vibrating blade microtome (Nussloch, Germany). Slices were immediately transferred to an interface tissue holding chamber and incubated for 1–2 h at room temperature in ACSF containing (in mm): 2 CaCl_2_, 1 MgCl_2_, 126 NaCl, 26 NaHCO_3_, 3.5 KCl, 1.26 NaH_2_PO_4_, 10 glucose). All the solutions were bubbled continuously with carboxygen (95% O_2_–5% CO_2_).

### Electrophysiology

Slices were placed in an interface recording chamber and perfused with warmed ACSF (2–3 ml min^−1^, driven by a peristaltic pump; Watson‐Marlow Pumps Ltd, Falmouth, UK, model 501U). The temperature of the chamber and perfusate was maintained at 33–36°C using a closed circulating heater Grant FH16D (Grant Instruments, Cambridge, UK). Extracellular field recordings were made using normal ACSF‐filled 1–3 MΩ borosilicate glass microelectrodes (GC120TF‐10; Harvard Apparatus, Camborne, UK) pulled using a Narishige electrode puller (PP‐83, Narishige Scientific Instruments, Tokyo, Japan), and mounted headstages (10× DC pre‐amp gain) held in Narishige YOU‐1 micromanipulators. In experiments involving dissections, scalpel blades were used to make cuts in the slices after placing them in the recording chamber. Waveform signals were acquired using BMA‐931 biopotential amplifier (Dataq Instruments, Akron, OH, USA), Micro 1401‐3 ADC board (Cambridge Electronic Design, Cambridge, UK) and Spike2 version 7.10 software (Cambridge Electronic Design). Signals were sampled at 10 kHz, amplified (gain: 200) and bandpass filtered (1–3000 Hz). A CED4001‐16 Mains Pulser (Cambridge Electronic Design) was connected to the events input of a CED micro 1401‐3 ADC board and was used to remove 50 Hz hum offline. Recordings were initiated while slices were still being perfused with normal ACSF, and only then was the perfusate switched to an epileptogenic ACSF solution lacking Mg^2+^ ions (0 Mg^2+^ACSF) or 100 μm 4‐aminopyridine (4‐AP). Hippocampal recordings were generally made from CA1, except for the baclofen experiments, where we made them from CA3, to provide a direct comparison with a previous study performed in rat (Swartzwelder *et al*. 1987).

Extracellular potassium (K^+^) was measured using single‐barrelled K^+^‐selective microelectrodes. The pipettes were pulled from non‐filamented borosilicate glass (Harvard Apparatus), and the glass was exposed to dimethyl‐trimethyl‐silylamine vapour (Sigma‐Aldrich, Gillingham, UK), baking at 200°C for 40 min, the pipettes were then backfilled with ACSF. A short column of the K^+^ sensor (Potassium Ionophore I, cocktail B; Sigma‐Aldrich, cat. no. 99373) was taken into the tip of the salinized pipette by using slight suction. The recordings through the K^+^‐sensor electrode were referenced to a second electrode filled with ACSF, and from the differential signal we calculated the [K^+^]_o_ from calibration recordings made in an open bath, using sudden increments in [K^+^]_o_. We checked the stability of the electrodes at the start and end of each recording. Data from unstable electrode recordings were discarded. This provided a scaling factor, *S*, of 55–59 mV, where the K^+^ concentration at a given moment in time, *t*, was calculated from the differential voltage, *V*(*t*), as follows:

K+o=K+o, baseline 10Vt/S

[K^+^]_o,baseline_ for our experiments was 3.5 mm.

### Data analysis and statistics

Data were analysed offline using Clampfit (Molecular Devices, Sunnyvale, CA, USA), Igor (WaveMetrics, Lake Oswego, OR, USA) and Matlab R2015b (The MathWorks, Natick, MA, USA). The analysis of entrainment of epileptiform events was performed by deconvolution of an averaged ‘template’ (Fig. 5 *D*) of electrophysiological discharges against the continuous trace from that same recording. In this way, the higher frequency components of these discharges are effectively removed. This is very helpful, because the cross‐correlation analyses between hippocampal and neocortical discharges is optimal if the signal can be simplified essentially to the timing of the events, thereby minimizing any aliasing issues that might arise from these higher frequency components. The deconvolution was done by first creating a template of an average discharge (6–10 events), aligned by the time point at which they exceeded a threshold set at between 25 and 40% of the peak deflection. The templates were then used as a normalizing filter on their respective raw traces, by deriving peak cross‐correlation coefficients for the time‐shifted template relative to the trace. This ‘template‐filtered’ trace (Fig. 5 *D*) removed most of the brain region‐specific fine structure of the individual discharges, but preserved their timing. Since the individual events in the late‐stage activity are extremely reproducible, the peaks in this filtered trace tend towards 1. We used the cross‐correlation between these template‐filtered recordings as a measure of the entrainment of the two recording locations. Matlab code for these analyses is available from the authors upon request. Percentage changes were measured by normalizing treatment to pre‐treatment measures in each slice. A one‐way analysis of variance (ANOVA), with a *post hoc* Tukey test was used for data with three or more groups. Groups of two were analysed using Student's *t* test. Data that were not normally distributed were analysed by the Wilcoxon rank sum test. Significance was set at *P* ≤ 0.05 for all analyses. Multiunit activity was extracted from raw data by high‐pass filtering it to >300 Hz. Data are presented as means ± SEM, and *n* is the number of brain slices, unless otherwise stated.

### Terminology

The terminology of epileptic discharges is problematic, reflecting the fact that there is a large range of activity patterns, and the equivalence of, or distinction between, these is often hard to discern. This is particularly so for the term ‘interictal’, which in the clinical setting refers to electrophysiological activity which is clinically covert (if not completely so; see Binnie *et al*. 1987; Kleen *et al*. 2010). These are typically rather short discharges, and consequently, animal researchers have taken their ephemeral nature to be the defining feature. Unfortunately when one examines the activity patterns closely, this term conflates two very different types of activity; an appreciation of the difference is critical for the understanding of the present study. The key distinction is the presence or absence of local intense activity, as defined by the presence of a significant high frequency component. The importance of this is that it helps distinguish sites where there is local pathological activity from those where the deviation in the recording reflects pathological activity that is elsewhere. Consequently, in this paper, we refer to the discharges during late stage activity as ‘spike and wave discharges’, a term that has been used previously to describe what appear to be comparable events. Readers should note, however, that previous studies have described this activity pattern as ‘interictal’, but for the reasons outlined above, we prefer not to use this term.

## Results

### Region‐specific patterns of evolving epileptiform activity

**Figure 1:**
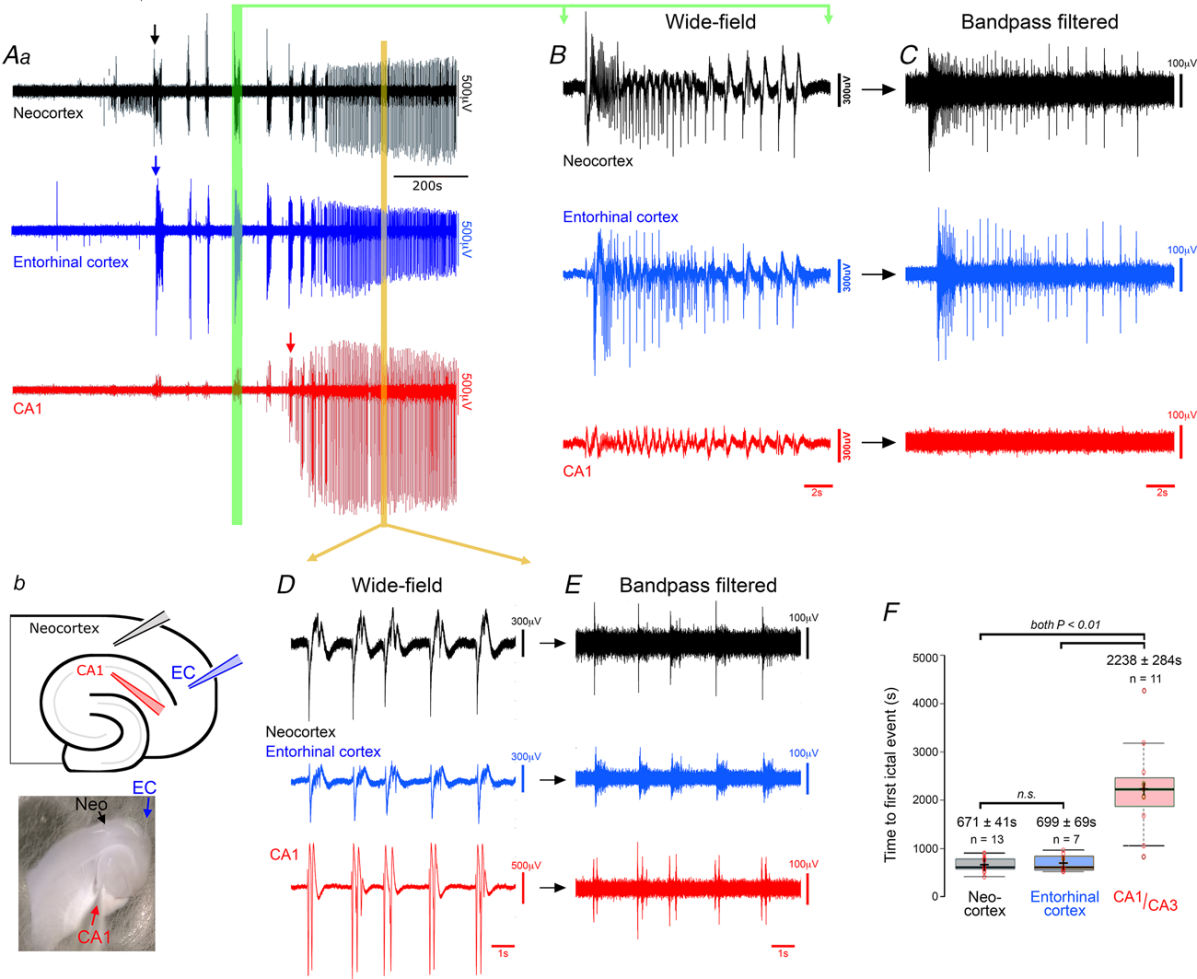
Typical pattern of evolving epileptiform activity following wash‐out of Mg^2+^ ions from the bathing media (0 Mg^2+^ model), showing delayed recruitment of hippocampal circuits relative to neocortex *Aa*, extracellular recordings (broad band), showing typical pattern of evolving epileptiform activity following washing out of Mg^2+^. The arrows indicate the first full ictal events, as indicated by intense multiunit (high frequency) activity, in the three recordings. *b*, schematic representation and photomicrograph of a horizontal brain slice, showing the locations of three extracellular recording electrodes in hippocampus (CA1, red), the entorhinal cortex (EC, blue) and deep layers of the neocortex (NC, black). *B*, broad band signals show small deflections in the hippocampal field at the time of large neocortical discharges during early ictal‐like events (green vertical bar in *A*), but high pass filtering (*C*) shows that these hippocampal signals are not associated with any significant unit activity. *D* and *E*, similar broad band (*D*) and high pass filtered (*E*) expanded views of a representative period of late‐stage activity (orange bar in panel *A*). Note that the prominent high frequency component indicative of local network firing, is now seen at all three electrode sites. *F*, boxplot illustrating the pooled data, showing a highly significant delay of the earliest hippocampal epileptiform discharges relative to the first neocortical or entorhinal discharges (ANOVA *F*_[2,28]_ = 25.76, *P* = 4.5 × 10^−7^). The results of individual comparisons (*post hoc* Tukey test) and the sample sizes (different brain slices; these are not paired recordings) are shown above the data distributions. [Color figure can be viewed at wileyonlinelibrary.com]

We investigated the evolution of epileptiform activity in horizontal brain slices, prepared from young adult (2–3 months old), wild‐type C57B6J mice, following the removal of Mg^2+^ ions from the bathing medium (ACSF). Extracellular recordings were made at two or three locations, always including a hippocampal (CA1 or CA3) and a neocortical (temporal association areas) recording site, and in most slices, also recording from medial entorhinal cortex (Fig. 1 *A*). Following the washout of Mg^2+^ ions, there was a gradual build‐up of epileptiform discharges, evolving in a highly characteristic way (Fig. 1 *A*). The earliest large field deflections in the raw traces were seen at all recording sites, although the events appeared far larger in the neocortex and entorhinal cortex. This early activity involved episodes of sustained rhythmic bursts suggestive of the temporal dynamics of clinical tonic–clonic discharges (Fig. 1 *B* and *C*). The mean number of tonic–clonic‐like events in the neocortex was 9.35 ± 0.73 per slice (range 4–17 events; *n* = 17 slices), before a second transition to regular epileptiform bursts (‘late‐stage activity pattern’), with individual bursts lasting a few hundred milliseconds, and occurring every 3.32 ± 0.38 s (*n* = 10; Fig. 1 *D* and *E*). This pattern of evolution has been described previously in rat brain slices (Swartzwelder *et al*. 1986a; Mody *et al*. 1987; Anderson *et al*. 1990; Dreier & Heinemann, 1990, 1991; Bragdon *et al*. 1992), but has been less studied in mice.

Recent studies of human extracellular recordings of epileptic discharges in humans have highlighted the importance of examining the high frequency component of epileptiform discharges to determine whether an event involves locally active neurons (Schevon *et al*. 2012; Weiss *et al*. 2013). In this regard, there appeared a striking difference between activity recorded in the hippocampus and the neocortical signals: the early events, including the tonic–clonic ictal events, were associated with only small field events in the hippocampus, and notably, with no measurable high frequency component (Fig. 1 *A–C*), indicating there is little local neuronal firing. We therefore considered these early events not to have invaded the local hippocampal networks. Using this high frequency component as the critical marker of ictal involvement, the first hippocampal ictal discharges occurred significantly later than the first neocortical discharges (Fig. 1 *A* arrows; Fig. 1 *F*; neocortex latency, 671 ± 41 s (*n* = 13); entorhinal cortex, 699 ± 69 s (*n* = 7); hippocampus, 2238 ± 284 s (*n* = 11); ANOVA *F*_[2,28]_ = 25.76, *P* = 4.5 × 10^−7^; neocortex *vs*. hippocampal, *post hoc* Tukey test, *P* = 0.001). Epileptiform discharges in entorhinal cortex evolved in tandem with the neocortical discharges (neocortex *vs*. entorhinal, not significant; entorhinal *vs*. hippocampal, *post hoc* Tukey test, *P* = 0.001).

When finally, the hippocampal epileptiform discharges began, they showed a fundamentally different pattern, generally being a single large spike and wave discharge lasting up to 1.26 ± 0.11 s (*n* = 10), or a short burst of discharges. In a further contrast to the prior neocortical activity, the inter‐event intervals were short (2.98 ± 0.78 s, *n* = 10), compared with the intervals between neocortical tonic–clonic ictal events (1st–2nd event interval = 126.2 ± 17.2 s; 2nd–3rd interval = 117.1 ± 15.1 s; 3rd–4th interval = 68.9 ± 10.6 s). Interestingly, the pattern of neocortical discharges also changed once the hippocampal discharges started, to the same pattern of transient, but regular, spike and wave discharges. Discharges in the two structures, from this time forward, were tightly coordinated (Fig. 1 *D* and *E*), but with the hippocampal discharges occurring before the neocortical unit activity (delay of onset of neocortical activity, relative to hippocampal activity = 87.1 ± 25.5 ms, *n* = 8).

**Figure 2:**
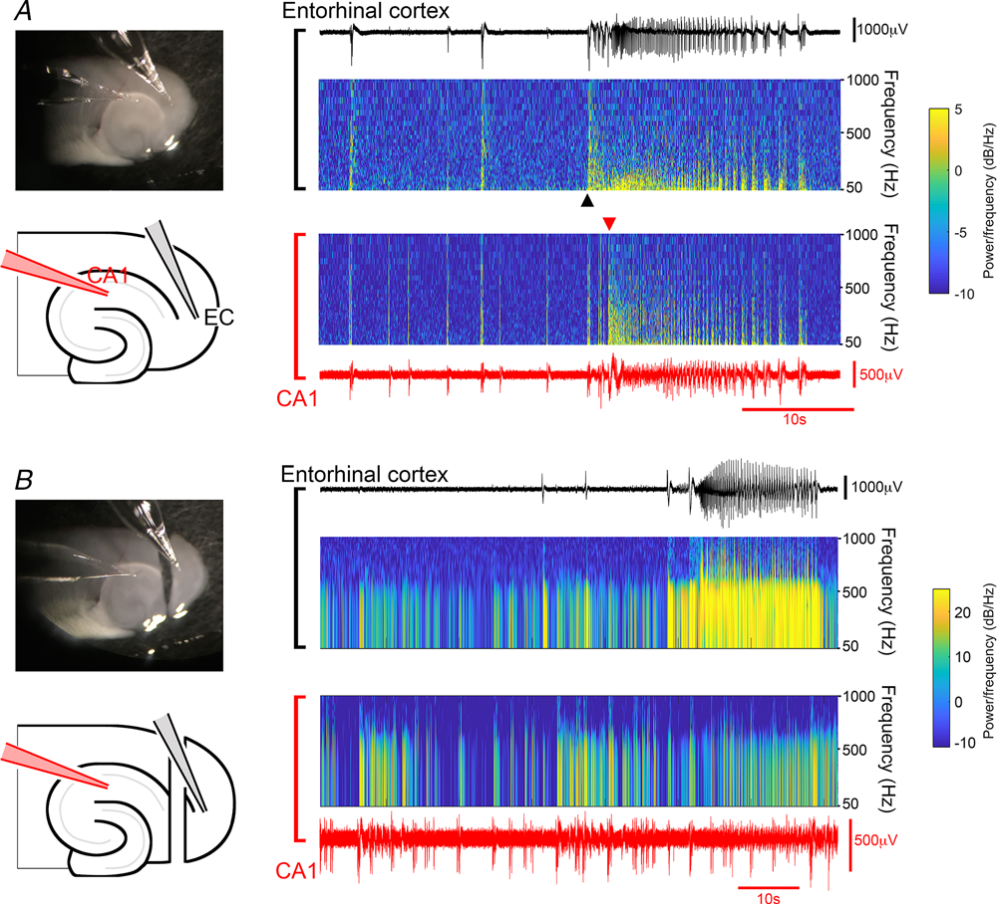
Tonic–clonic activation of CA1 can spread from entorhinal cortex *A*, ventral horizontal brain slice, in which tonic–clonic activity spreads from entorhinal cortex into CA1. The spectrograms show that the sustained (tonic) high frequency component occurs first in the entorhinal cortex. *B*, recording from the same slice, following sectioning of the temporoammonic pathway. Note the tonic–clonic‐like event in entorhinal cortex, but not in CA1. [Color figure can be viewed at wileyonlinelibrary.com]

A noteworthy feature of these recordings was that tonic–clonic discharges appeared to be a hallmark only of neocortical recordings, with repeated events in every recording (*n* = 13). In contrast, we recorded such events in hippocampal electrodes in just 7.7% of the slices (1 in 13 recordings). There are, however, published records from rat brain slices of hippocampal tonic–clonic ictal events (Swartzwelder *et al*. 1987; Lewis *et al*. 1989), but using much thicker brain slices (625 μm). A key question then was whether this represented a species difference, or if instead, the thicker brain slices showed hippocampal tonic–clonic events because of better preserved neuronal connectivity. Our recordings were made typically from the middle sections in the dorsal–ventral axis, but we reasoned that on account of the curvature of the hippocampus, other levels may show different preservation of the axonal pathways. We therefore examined the more extreme slices, and discovered that the most ventral mouse brain slices (400 μm, *n* = 4 slices) also showed tonic–clonic activity in CA1 (Fig. 2 *A*), as seen in thick (>600 μm), rat sections; in this regard, therefore, there is no species difference. Notably, the tonic–clonic activity occurred in the entorhinal cortex before the CA1 region (Fig. 2 *A*), suggestive that the CA1 activity was conditional on the entorhinal cortex activity. This was confirmed by separating the two structures, after which the tonic–clonic pattern was maintained in entorhinal cortex, but abolished in CA1 (Fig. 2 *B*), which instead resorted to the spike and wave events already described. We conclude therefore that the early pattern of tonic–clonic activity is a hallmark of neo‐ and entorhinal cortex, and that instances of such activation in the hippocampus are downstream of activity at these other sites.

### Non‐canonical propagation pattern of late epileptiform discharges

In contrast, during late stage activity, the hippocampal activity appears to be the pacemaker, entraining the other areas. We hypothesized that the entrainment is mediated through a polysynaptic pathway involving the entorhinal cortex. To test this, we cut away the caudal pole of the brain slice (we refer to these, henceforth, as ‘disconnected slices’), thereby entirely removing any potential synaptic pathway. Surprisingly, following the removal of the entorhinal pole, the hippocampal entrainment of neocortical discharges persisted unchanged (neocortex, pre‐cut rate = 0.47 ± 0.08 Hz, post‐cut = 0.47 ± 0.09 Hz, *n* = 5, paired *t* test, *P* = 0.96; hippocampus, pre‐cut rate = 0.48 ± 0.09 Hz, post‐cut = 0.49 ± 0.11 Hz, *n* = 5, paired *t* test, *P* = 0.83), and latency of onset of neocortical activity after hippocampal activity also remained unaltered (pre‐cut = 71.1 ± 7.7 ms, post‐cut = 62.4 ± 3.6 ms, *n* = 5, paired *t* test, *P* = 0.13).

**Figure 3:**
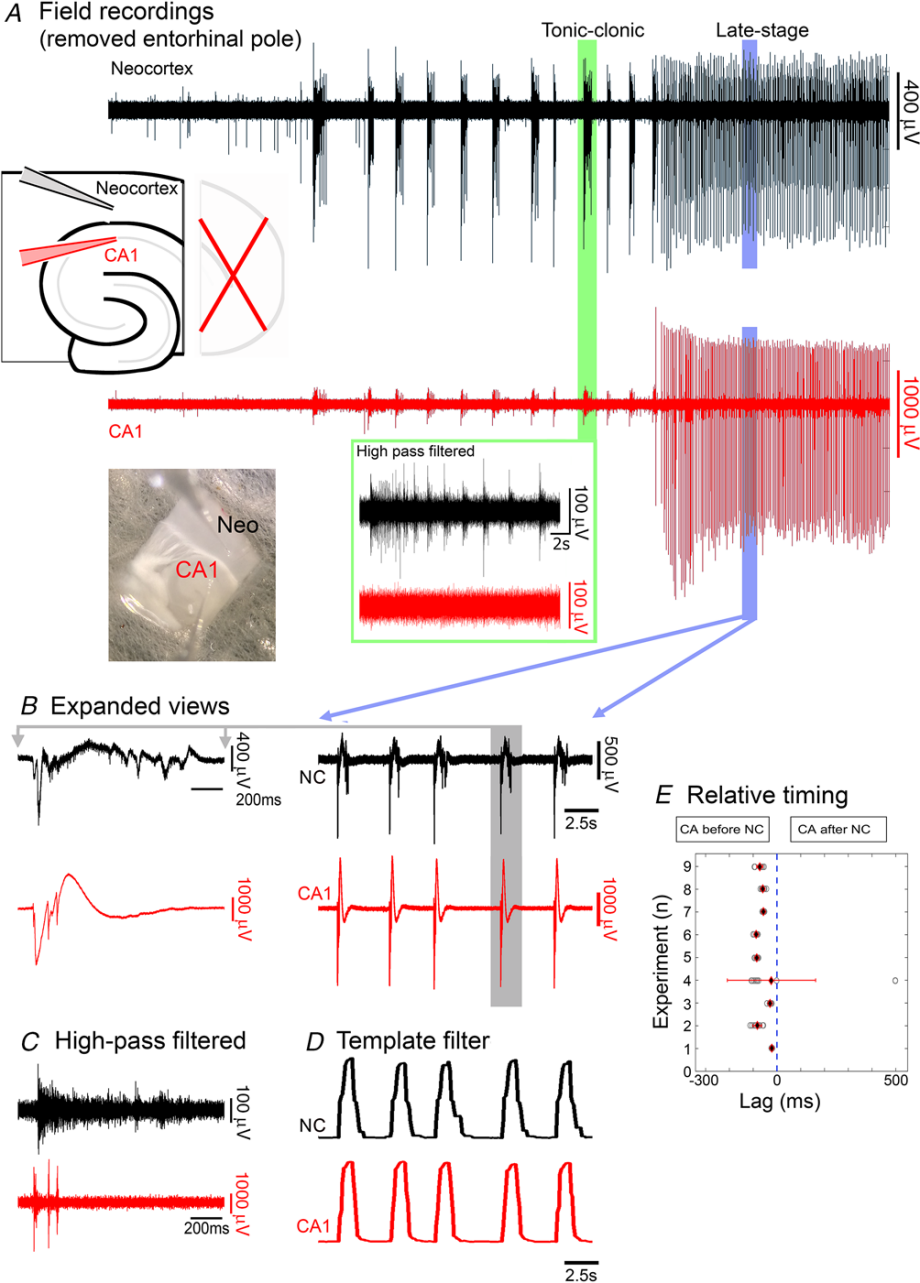
The late‐stage epileptiform discharges are coordinated in hippocampal and neocortical networks through a non‐synaptic pathway *A*, extended recording of extracellular field potentials in CA1 and neocortex (NC), following wash‐out of Mg^2+^, in a disconnected slice, i.e. with entorhinal cortex removed, thereby disconnecting the two regions via any conventional multisynaptic path. As in intact slices, the early discharges showed pronounced unit activity in neocortex, but not in the CA1 pyramidal layer (inset, green box). *B* and *C*, expanded view of wide‐band (*B*) and high‐pass (>300 Hz) filtered (*C*) late stage activity in the same slice, showing prominent levels of unit activity in both territories. *D*, the same traces filtered by a moving template of an average discharge. *E*, graphical representation of data from 9 brain slices, showing that in synaptically disconnected, hippocampal–neocortical slices, late‐stage discharges in neocortex (NC) follow hippocampal (CA) discharges (average lag = 57.8 ± 9.1 ms; one‐sample *t* test: *P* = 0.0002, *n* = 9). [Color figure can be viewed at wileyonlinelibrary.com]

**Figure 4:**
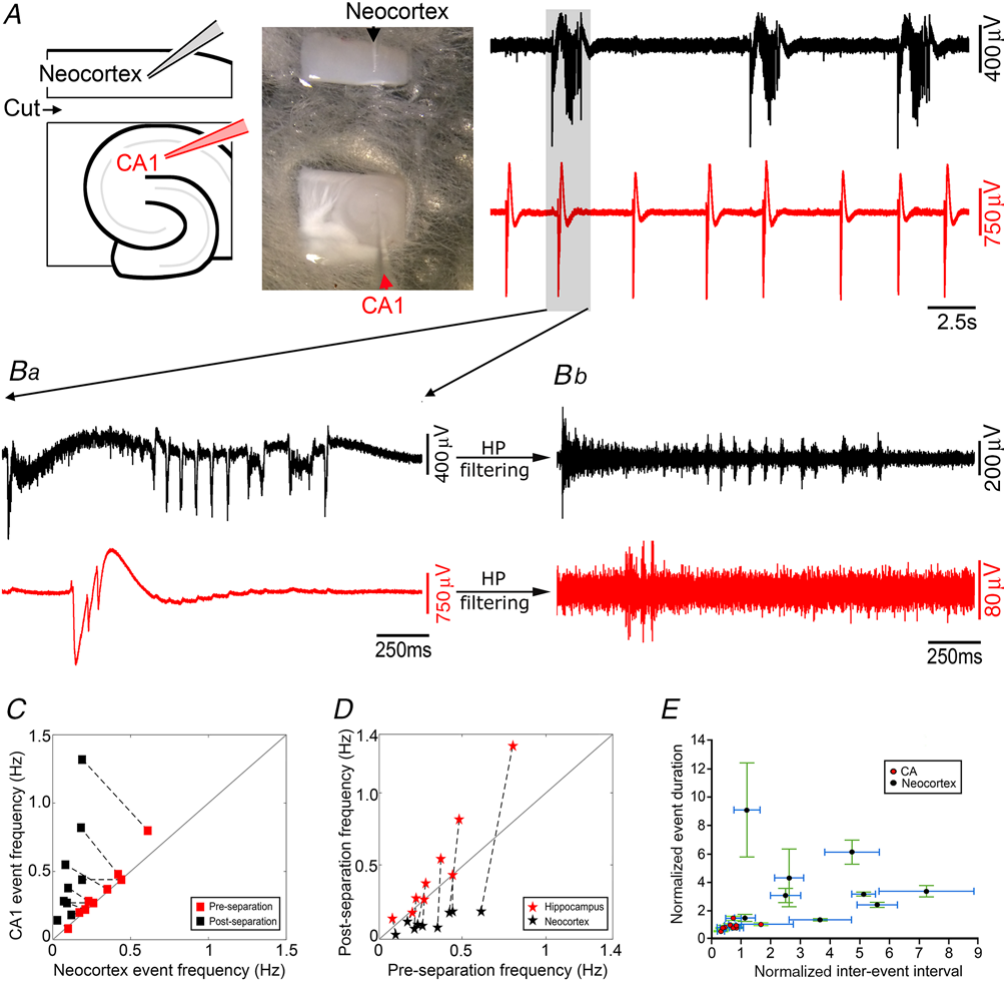
Entrainment of discharges is lost following physical separation of hippocampal and neocortical networks *A*, photomicrograph and schematic representation showing the electrode placements in physically separated CA1 and neocortical (NC) areas, derived from a single horizontal brain slice, together with a period of late stage epileptiform discharges. Note the desynchronized discharges in the two territories, with a far slower rate of discharges in the neocortical tissue. *Ba*, further expansions show the broadband signal of the de‐synchronized hippocampal and neocortical discharges. *b*, prominent unit activity is seen in both territories. *C*, the relative rates of epileptiform discharges in the two territories before and after physical separation. In disconnected slices (‘pre‐separation’), the rates were equivalent (n.s., *n* = 9), but following physical separation of the tissues, the rates are significantly different (paired *t* test, *P* = 0.011, *n* = 9). *D*, comparisons of discharge rates before and after physical separation of the hippocampal and neocortical tissues. Note how the neocortical data all fall below the line of unity, indicating a consistent slowing of the rate of discharges there (black stars; paired *t* test, *P* = 0.001, *n* = 9). In contrast, the hippocampal data tend to lie above the line, indicative of an increase in hippocampal rate after the separation (red stars; paired *t* test, *P* = 0.045, *n* = 9). *E*, the duration and inter‐event intervals in the physically separated hippocampal and neocortical tissues, normalized to the values in the pre‐cut brain slice. (Error bars depict the standard deviation.) [Color figure can be viewed at wileyonlinelibrary.com]

In slices with the entorhinal pole removed from the start of the experiment (simultaneous with washing out Mg^2+^ ions), the evolving epileptiform activity showed the same general pattern in ‘intact slices’ (slices including the entorhinal pole), albeit at a slightly slower rate (Fig. 3 *A*; neocortex latency, 1035.71 ± 77.8 s, *n* = 14; intact *vs*. disconnected, unpaired *t* test, *P* = 0.0006). The first hippocampal ictal discharge occurred significantly later than the first neocortical discharge (hippocampus latency, 2356.10 ± 189.10 s, *n* = 14; latency: hippocampus *vs*. neocortex, paired *t* test, *P* = 0.0004; hippocampus latency, intact *vs*. disconnected, unpaired *t* test, *P* = 0.79). And as with the intact slices, the start of the hippocampal discharges entrained the neocortical activity to the same pattern, despite the absence of any conventional polysynaptic connectivity between the two regions (Fig. 3 *B–E*; neocortical latency, 57.8 ± 9.1 ms, one‐sample *t* test, *P* = 0.0002; intact *vs*. disconnected, unpaired *t* test, *P* = 0.27). This entrainment was only lost when a second cut was made along the axis of the white matter bundle deep to the neocortical layer 6, thereby physically separating the neocortical and hippocampal networks (Fig. 4). In these separate networks, the hippocampal discharge rate increased significantly (pre‐cut, 0.35 ± 0.07 Hz; post‐cut, 0.49 ± 0.13 Hz; *n* = 9, paired *t* test, *P* = 0.045; Fig. 4 *C–E*), whereas the neocortical discharge rate dropped significantly (pre‐cut, 0.31 ± 0.05 Hz; post‐cut, 0.12 ± 0.02 Hz; *n* = 9, paired *t* test, *P* = 0.001; Fig. 4 *C–E*). Consistent with these respective changes, the rate of discharge in the isolated neocortex differed significantly from that in the isolated CA territories (post‐cut; paired *t* test, *P* = 0.011). In tandem with the reduced rate of discharges in the neocortical networks, the duration of events became longer (pre‐cut = 1.82 ± 0.21 s, post‐cut = 6.70 ± 2.19 s, *n* = 9, paired *t* test, *P* = 0.048; Fig. 4 *E*), and again there was a significant difference between the isolated neocortex and hippocampus (post‐cut, *n* = 9; paired *t* test, *P* = 0.041). This result suggests that, in this late stage activity pattern, the interactions between hippocampal and neocortical networks are bidirectional: the hippocampal‐to‐neocortical influence is reflected in the pacing of neocortex by hippocampus; whereas the opposite influence is manifest as a mild brake on the hippocampal pacing, presumably by the tendency of the neocortical events to be extended, thereby also extending the refractoriness of the hippocampal pacemaker.

Consistent with these opposite changes in rates, there was a highly significant drop in the correlation of events in the two networks (*n* = 9 brain slices; pre‐cut *R* ^2^ range = 0.83–0.98; post‐cut *R* ^2^ range = 0.33–0.58; *P* = 4.1 × 10^−5^; Wilcoxon rank sum test). We concluded from these experiments that the late stage epileptiform discharges arise in hippocampus, and these act as a pacemaker, driving discharges also in juxtaposed neocortical territories, and that this entrainment can occur independent of synaptic interactions (importantly, note that it does not exclude the possible involvement of conventional synaptic pathways in the development and propagation of epileptiform activity).

The interactions between the areas only really affected the late stage activity, because in slices that were dissected at the start of the experiment, to isolate the neocortex, entorhinal cortex and hippocampal subfields, the time to first ictal events in all three territories was unaltered, relative to recordings from intact slices (isolated neocortex, 607.3 ± 107.3 s, *n* = 6, unpaired *t* test *vs*. intact, *P* = 0.504; entorhinal cortex, 1057.2 ± 200.1, *n* = 5, unpaired *t* test *vs*. intact, *P* = 0.082; hippocampus, 1595.4 ± 186.3, *n* = 6, unpaired *t* test *vs*. intact, *P* = 0.140).

**Figure 5:**
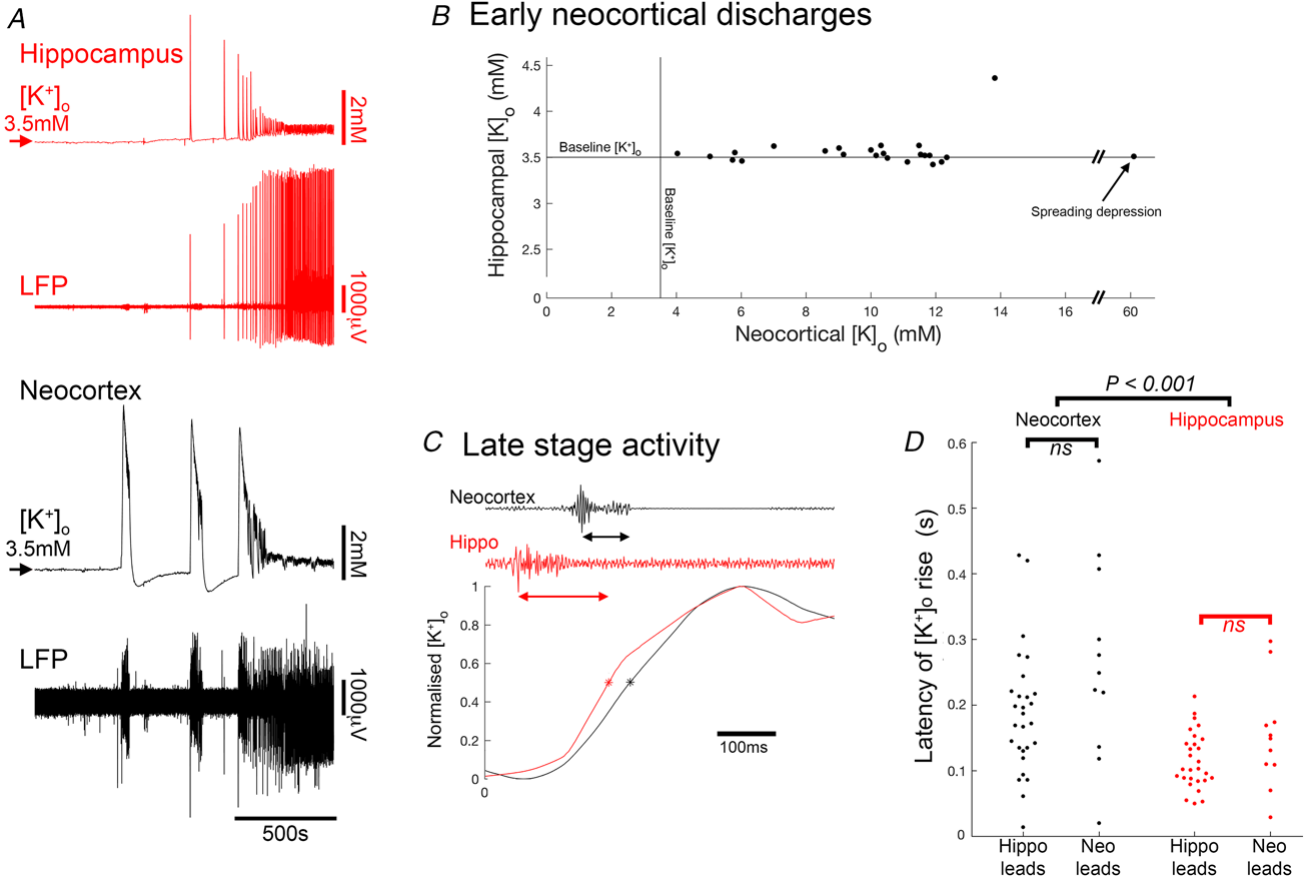
Entrainment does not happen through diffusion of K^+^ *A*, concurrent recordings of LFPs and [K^+^]_o_ in CA1 and neocortex. *B*, peak transient [K^+^]_o_, and the CA1 [K^+^]_o_ measurements, during early neocortical tonic–clonic epileptiform events when there was no corresponding multiunit activity in CA1. *C*, latency measurements during late stage events, with multiunit activity occurring in both neocortex and CA1 regions. *D*, population data, for the latency measurements (41 data points from 6 brain slices) segregated by whether the neocortex or the hippocampal activity came first (unpaired *t* test; *P* = 0.00015). [Color figure can be viewed at wileyonlinelibrary.com]

One possible mechanism by which late‐stage entrainment may happen is through diffusion of extracellular K^+^ from a local source of intense neuronal activation (Moody *et al*. 1974; Heinemann & Lux, 1977; Somjen & Giacchino, 1985; Hablitz & Heinemann, 1987), thereby reducing the threshold for recruitment of other neighbouring territories. To assess the effects of [K^+^]_o,_ rises, we made simultaneous recordings from four electrodes, at two sites, two located in neocortex and two in the CA1 region of the hippocampus, to record the local [K^+^]_o_, using an ionophore tip‐filled electrode, and the local field potential (LFP). We found that the largest rises in [K^+^]_o_ associated with epileptiform events all occurred during the early tonic–clonic events in neocortex (Fig. 5; note that much larger rises were, on occasion, recorded during spreading depression events, but these did not show high frequency activity denoting local neuronal firing). We analysed 24 events, in six brain slices (intact, not disconnected), for which the multiunit activity showed that the event only occurred at one electrode site (Fig. 5; 23 neocortical events, and 1 hippocampal discharge). Critically, in all cases, the site of unit activity was associated with a large rise in [K^+^]_o_ (neocortical examples (*n* = 23), [K^+^]_o_ = 9.55 ± 2.70 mm), but this did not spread to the other recording site (hippocampal [K^+^]_o_ = 3.57 ± 0.18 mm; not significantly different from baseline [K^+^]_o_ = 3.50 mm; Fig. 5 *A* and *B*). This was also the case for the single example of a prominent hippocampal discharge without neocortical involvement (hippocampal [K^+^]_o_ = 7.26 mm; neocortical [K^+^]_o_ = 3.13 mm). This showed that the [K^+^]_o_ changes are highly focal, indicating that this entrainment at a distance is not mediated by diffusion of K^+^.

We next examined the late stage activity in which there was multiunit activation at both neocortical and hippocampal locations (41 events from 6 slices; Fig. 5 *C* and *D*). As expected, both sites also showed significant rises in [K^+^]_o_ associated with these bursts of local neuronal firing, and in all cases the main rise came after the local peak in the high frequency filtered LFP signal. There are, however, inherent problems with comparing timing between high and low bandpass‐filtered signals, so we performed a further analysis comparing the latency of the rise in [K^+^]_o_ between those events which lead in the hippocampal electrode (*n* = 30), and those that led in the neocortical electrode (*n* = 11). We reasoned that if activity in the follower territory was being triggered by a rise in [K^+^]_o_ diffused from the other site, then for those events, the rise would appear to occur significantly earlier relative to the local firing. In fact, there was no significant difference in latency between ‘leader’ and ‘follower’ events for either the hippocampal or the neocortical recording sites (Fig. 5 *D*), leading us to conclude that in both groups, the local [K^+^]_o_ rise reflected, rather than caused, the local firing. We did observe that the latency for the [K^+^]_o_ rise in hippocampal circuits was significantly shorter than for neocortical circuits (unpaired *t* test; *P* = 0.00015), perhaps reflective of more densely packed neurons in the hippocampus. Collectively, these various analyses of [K^+^]_o_ rises associated with local neuronal firing indicate that the entrainment of neocortical events by the hippocampal activity in this preparation does not happen by diffusion of K^+^ ions. Instead, it is likely to occur by the distant effects of a field potential onto circuits that are already highly excitable. This type of entrainment, we suggest, is also possible *in vivo*, during clinical epileptic events.

### Region‐specific differences in drug sensitivity influence epileptic activity patterns

**Figure 6:**
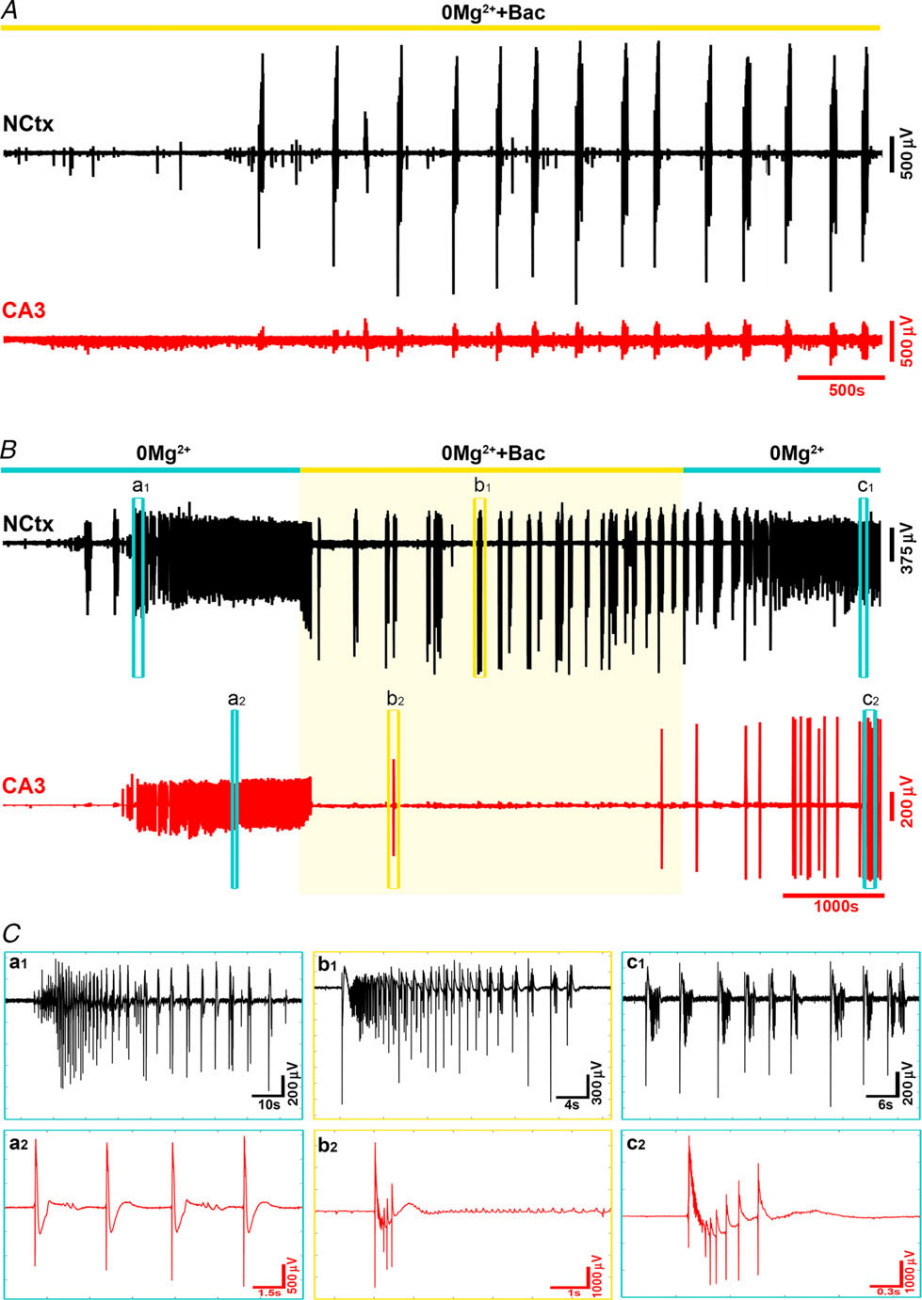
Hippocampal, but not neocortical epileptiform activity, is suppressed by GABA_B_ activation *A*, the GABA_B_ agonist baclofen, when applied simultaneously with the wash‐out of Mg^2+^ ions, blocks any developing hippocampal activity, but does not suppress the development of tonic–clonic‐like events in neocortex. *B*, baclofen also blocks the hippocampal activity after it has started, thereby reversing the late stage pattern, and initiating the tonic–clonic‐like events that characterize the neocortical pattern. *C*, enlarged views of the discharges at the times indicated in *B*. [Color figure can be viewed at wileyonlinelibrary.com]

Previous work suggests that the different phases of evolving activity in this model activity may show differential sensitivity to drug manipulation. We investigated whether this may relate to regional sensitivity. One promising candidate is the GABA_B_ agonist, baclofen, which was reported to reverse the evolving pattern of activity, inducing a switch from what we term late stage activity (Swartzwelder *et al*. 1987; Lewis *et al*. 1989) (in the original description this was termed ‘interictal’ activity; see Methods, Terminology) into tonic–clonic events. These experiments were performed on thick (600 μm) rat brain slices, but since the activity generalized throughout the slice, the authors of the previous studies did not relate this to the source of activity. We repeated these experiments therefore on 400 μm mouse brain slices to investigate whether the effect was location specific (Fig. 6; in keeping with these prior studies, we recorded from CA3 in these experiments). We found that bath application of the GABA_B_ agonist baclofen (10 μm) did indeed reverse the late‐stage pattern (4 out of 4 slices), suppressing entirely the hippocampal bursting, and with the reappearance of tonic–clonic events in neocortex (Fig. 6 *B*). Furthermore, if baclofen were applied from the start of the recording (when washing out Mg^2+^ ions), the tonic–clonic epileptiform events took longer to establish (0 Mg^2+^ latency = 609 ± 31 s (*n* = 5 slices); baclofen with 0 Mg^2+^ latency = 1573 ± 176 s (*n* = 4); unpaired *t* test, *P* = 0.0005), but once that happened were maintained for the entire duration of the recordings, and hippocampal discharges never initiated (Fig. 6 *A*). These results further support our conclusion that the different patterns of epileptiform bursting appear pathognomonic of the territories from which they originate.

**Figure 7:**
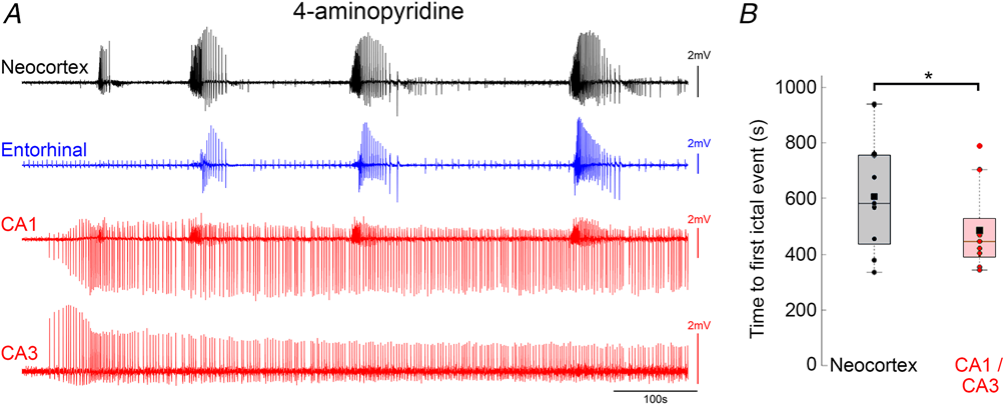
4‐Aminopyridine activates hippocampal networks earlier than neocortical networks *A*, quadruple electrode recording, showing the early activation of CA1 and CA3 in advance of neocortical epileptiform activation. Note that again the CA1 territory in this recording shows some tonic–clonic‐like activity that reflects the input from entorhinal cortex. *B*, pooled data showing that hippocampal discharges in this mode precede those in neocortex (paired *t* test, ^*^ *P* = 0.04). [Color figure can be viewed at wileyonlinelibrary.com]

**Figure 8:**
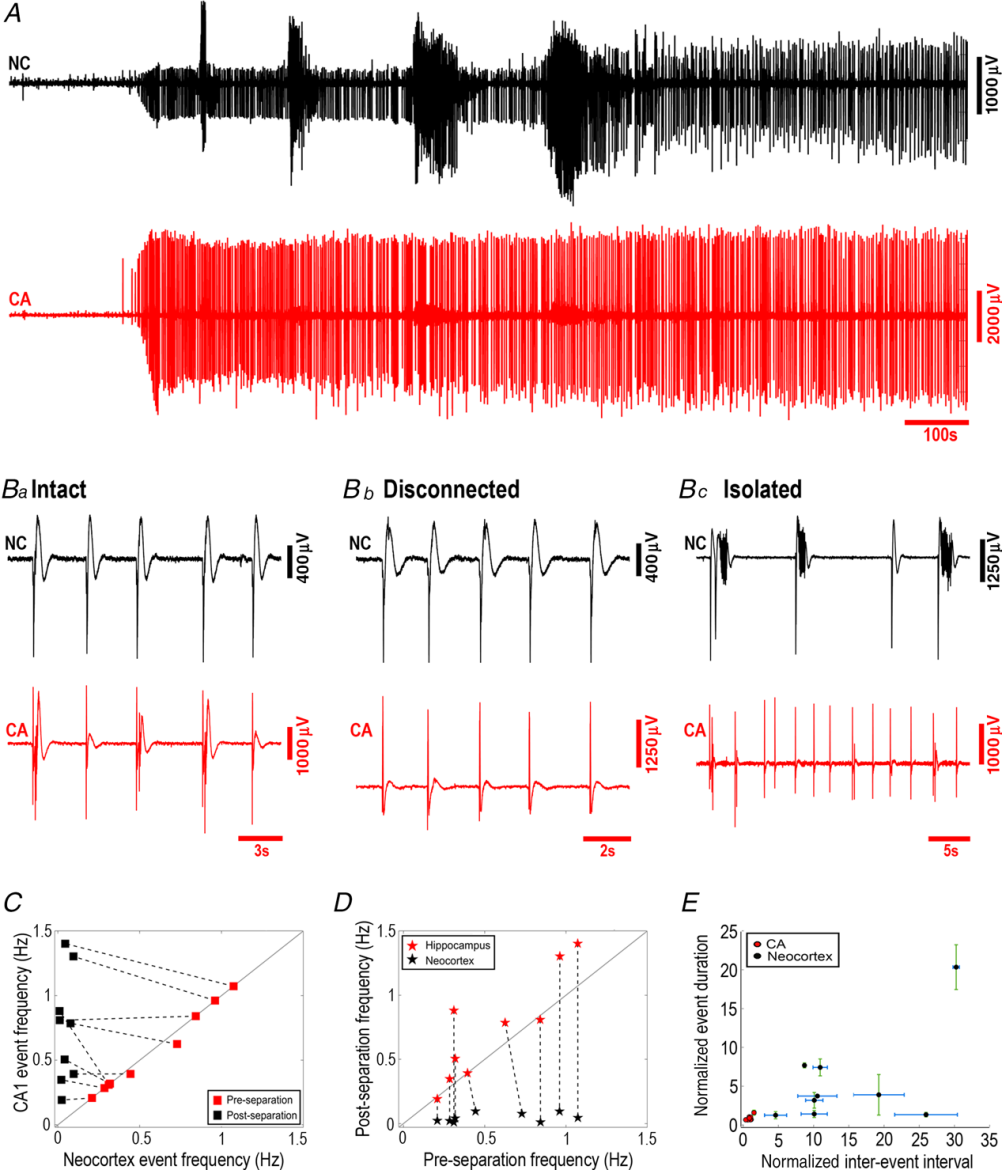
Entrainment patterns in 4‐aminopyridine are comparable to those in 0 Mg^2+^ *A*, a paired field recording from neocortex (black) and CA, showing the evolving pattern of epileptiform discharges when the slice was bathed in 4‐aminopyridine. *B–D*, activity was entrained in the intact slices (*Ba*), and persisted after removal of the entorhinal pole (*Bb* and *C*), but was lost after separation of the neocortex and CA territories (*Bc*). *C*, relative rates of epileptiform discharges before and after physical separation of the hippocampal and neocortical regions. In disconnected slices (‘pre‐cut’), the rates were similar in both regions (n.s., *n* = 9), but following physical separation (‘post‐cut’) the rates were significantly different (paired *t* test, *P* = 0.0011; *n* = 9). *D*, comparison of discharge rates before and after physical separation of the neocortical and hippocampal regions, showing a change in both neocortex (black stars, paired *t* test, *P* = 0.0011, *n* = 9) and CA1 (red stars, paired *t* test, *P* = 0.0301, *n* = 9). *E*, event duration and inter‐event intervals in post‐separated hippocampal and neocortical tissues normalized to the values in pre‐separated slices. (Error bars depict the standard deviation.) [Color figure can be viewed at wileyonlinelibrary.com]

This region‐specific difference also has a parallel in another under‐appreciated feature of brain slice models, which is that application of the K^+^ channel blocker 4‐aminopyridine has the exact opposite regional specificity to the 0 Mg^2+^ model, inducing epileptiform discharges in hippocampal territories significantly in advance of that in neocortex (Fig. 7; neocortical latency = 605 ± 22 s (*n* = 9 slices); CA3 latency = 487 ± 17 s (*n* = 9); paired *t* test, *P* = 0.04). Notably, the early hippocampal activity in this instance does not typically entrain the neocortical territories, indicating that this entrainment requires changes in the local excitability. Later, though, hippocampal entrainment does occur (Fig. 8). Entrainment between hippocampal territories and neocortex was not altered by removal of the entorhinal pole (neocortical latency: intact = 45.1 ± 8.2 ms, disconnected = 39.3 ± 2.8 ms, *n* = 8, paired *t* test, *P* = 0.44), but as with the 0 Mg^2+^ model, entrainment was broken by physically separating the hippocampal territories from neocortex (Fig. 8 *B* and *D*), leading once again to a slowing of the neocortical rhythm (pre‐cut, 0.57 ± 0.10 Hz; post‐cut, 0.05 ± 0.01 Hz; *n* = 9; paired *t* test, *P* = 0.0011; Fig. 8 *C–E*), and an increase in the hippocampal rhythm (pre‐cut, 0.56 ± 0.10 Hz; post‐cut, 0.74 ± 0.13 Hz; *n* = 9, paired *t* test, *P* = 0.0301). Consistent with these changes, there was also a significant difference between the isolated neocortex and isolated hippocampal CA territories (post‐cut, *n* = 9; paired *t* test, *P* = 0.0011, Fig. 8 *C–E*). There was also a trend towards an increase in duration of events in neocortex (pre‐cut, 1.23 ± 0.11 s; post‐cut, 6.23 ± 1.61 s; *n* = 9; paired *t* test, *P* = 0.0161; Fig. 8 *E*), but not in the hippocampus (pre‐cut, 1.17 ± 0.11 s; post‐cut, 0.97 ± 0.06 s; *n* = 9; paired *t* test, *P* = 0.106; Fig. 8 *E*). The isolated neocortex showed significantly longer events than the isolated hippocampus (post‐cut, *n* = 9; paired *t* test, *P* = 0.0147). In all these features, the 4‐aminopyridine and the 0 Mg^2+^ models were comparable.

**Figure 9:**
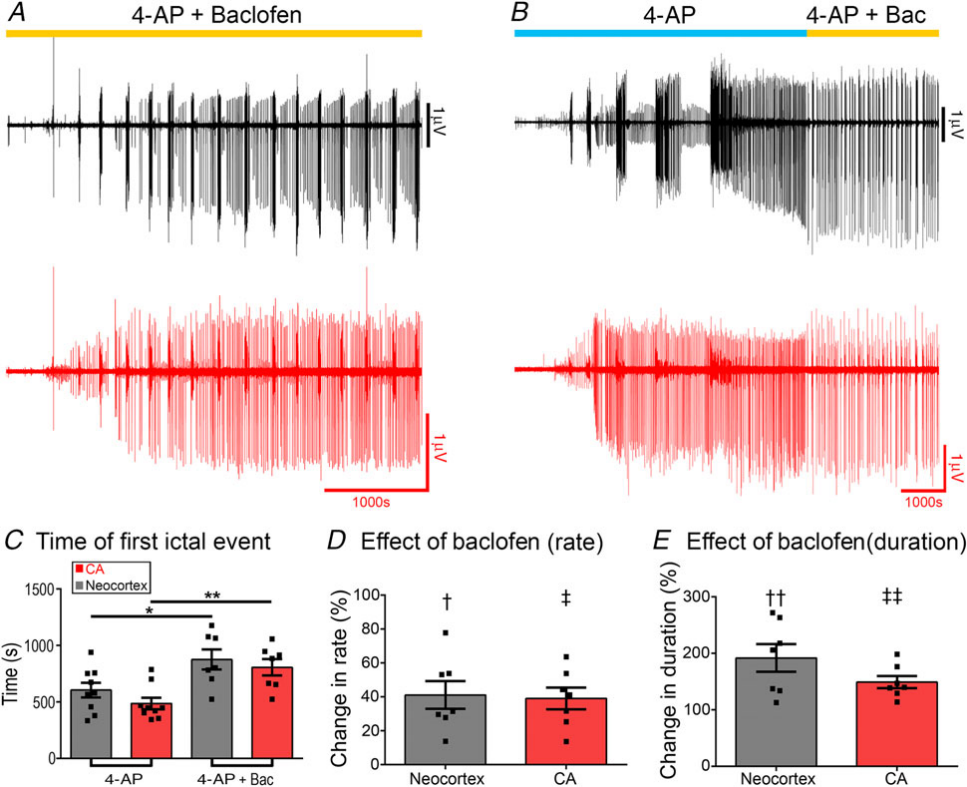
Baclofen has a smaller effect on 4‐aminopyridine‐induced activity than it does on 0 Mg^2+^‐induced activity *A*, prolonged (>1 h) recording of a brain slice bathed in both baclofen and 4‐aminopyridine (4‐AP) from the start of the recording. *B*, recording of a brain slice in which baclofen was applied only after the late‐stage activity pattern was reached. The activity was partially reversed, although unlike the 0 Mg^2+^ there was continued hippocampal discharges. *C*, baclofen, when applied from the start of the experiment, delayed the time to the earliest ictal events in both neocortex and hippocampus (protocol as in *A*; ANOVA *F*_[3 28]_ = 6.75, *post hoc* Tukey test: ^*^ *P* = 0.046; ^**^ *P* = 0.014). *D* and *E*, in both neocortex and CA, baclofen induced a reduction in the rate of the late stage discharges (*D*; protocol as in *B*; † paired *t* test, *P* = 0.012; ‡ Wilcoxon paired rank test, *P* = 0.016) and a marked increase in the duration of events (*E*; †† paired *t* test, *P* = 0.016; ‡‡ paired *t* test *P* = 0.002). [Color figure can be viewed at wileyonlinelibrary.com]

Finally, we examined the effect of baclofen on the activity patterns in 4‐aminopyridine (Fig. 9). When baclofen was applied from the start of the recording, simultaneous with the wash‐in of 4‐aminopyridine, full ictal activity evolved in the neocortical structures, and in 2 of 7 slices (28.6% of slices) it persisted throughout the recording, with no transition to late‐stage activity patterns (Fig. 9 *A*). Baclofen delayed the onset of hippocampal discharges (Fig. 9 *C*; latency in 4‐AP = 487 ± 51 s (*n* = 9), in 4‐AP with baclofen = 807 ± 73 s (*n* = 7); ANOVA *F*_[3,28]_ = 6.75, *P* = 0.001; *post hoc* Tukey test, *P* = 0.014), and also the first neocortical discharges (Fig. 9 *C*; latency in 4‐AP = 605 ± 65 s (*n* = 9); in 4‐AP with baclofen = 876 ± 89 s (*n* = 7); *post hoc* Tukey test, *P* = 0.046). When baclofen was applied after the late‐stage activity evolved, it caused a partial reversal of activity, with a reduction in the rate of discharges (neocortex: 4‐AP, 0.31 ± 0.05 Hz; 4‐AP+baclofen, 0.11 ± 0.02 Hz; percentage change (4‐AP = 100%) = 41.1 ± 8.2% (*n* = 7); paired *t* test, *P* = 0.012; hippocampal: 4‐AP, 0.32 ± 0.06 Hz; 4‐AP+baclofen, 0.12 ± 0.03; percentage change (4‐AP = 100%) = 39.1 ± 6.4% (*n* = 7); Wilcoxon paired rank test, *P* = 0.016), and a lengthening of the discharge duration (neocortex: 4‐AP, 1.37 ± 0.08 s; 4‐AP+baclofen, 2.72 ± 0.47 s; percentage change (4‐AP = 100%) = 191.9 ± 24.3% (*n* = 7); paired *t* test, *P* = 0.016; hippocampal: 4‐AP, 1.26 ± 0.10 s; 4‐AP+baclofen, 1.86 ± 0.16 s; percentage change (4‐AP = 100%) = 149.2 ± 10.9% (*n* = 7); paired *t* test *P* = 0.002), but it neither fully blocked the hippocampal nor neocortical activity (Fig. 9 *B*, *D* and *E*). Thus, in 4‐aminopyridine, the baclofen partially reproduced the effects seen in the 0 Mg^2+^ model.

In summary, we conclude that certain electrophysiological transitions arise from switches in the source of the pathological discharges, and reflect brain region‐specific differences in the propensity to support epileptiform discharges, and the electrophysiological signatures of these discharges. These switches between the focal sources can occur spontaneously, presumably reflecting local or cellular changes in the network excitability, but can also be influenced pharmacologically, indicating brain region‐specific differences, too, in their drug sensitivity.

## Discussion

We have provided demonstrations for several key principles of epileptic pathophysiology. The first is that transitions in the pattern of activity can reflect shifts in the source of discharges. A second key finding is that brain regions differ in how epileptic discharges manifest in the local circuits. A third principle is that when cortical networks have raised levels of excitability, they can be entrained through non‐synaptic paths. These conclusions arose because there appear to be very considerable differences between the patterns of epileptic discharges in neocortex and the CA territories. Entorhinal cortex appears to follow the neocortical pattern, while the different CA territories appear broadly similar, although we do not discount the possibility that there may yet be subtle differences between these.

It is important to realize that these are not the only changes underlying the development of epileptic activity (Whittington *et al*. 1995; Fujiwara‐Tsukamoto *et al*. 2007; Ellender *et al*. 2014). However, these various experiments do provide evidence of the interesting interplay between areas that are driving the pathology (the source, or ictal focus) and the susceptibility of secondary territories to be recruited. Of course, these acute brain slice preparations clearly do not incorporate all facets of the epileptic condition, but the substrates for all three of these principles do exist *in vivo*, and so, we would argue, all are likely to be relevant in spontaneously occurring seizures in humans.

The main focus of our studies was the marked transition from early tonic–clonic activity to a late‐stage pattern of repeated, spike and wave discharges, occurring every 2–10 s typically. This builds upon previous work done mainly using rat brain slices (Swartzwelder *et al*. 1986b; Mody *et al*. 1987; Anderson *et al*. 1990; Dreier & Heinemann, 1990, 1991; Bragdon *et al*. 1992; Morrisett *et al*. 1993; Zhang *et al*. 1995; Dreier *et al*. 1998), but we extend this in two important ways. The first is that, with the development of various mouse models carrying genetic mutations associated with human epileptic conditions (Yu *et al*. 2006; Asinof *et al*. 2015), our studies provide important confirmation that the evolving activity patterns in these models follow the same pattern in mice as they do in rats. This will facilitate a productive line of investigations regarding exactly how specific genetic mutations impact on network stability, using the 0 Mg^2+^ and 4‐AP models. Since these two models induce distinct, and yet highly characteristic, activity patterns in brain slices from normal cortex, we expect that when these same models are applied to slices from epileptic, transgenic animals, they would provide indications of exactly where in the network the transgene has its effect. We described this approach as a kind of ‘stress‐test’, to understand how genetic mutations alter network performance (Parrish & Trevelyan, 2018).

The second advance has been to clarify that different brain territories sustain characteristic epileptic discharge patterns. The extension of cortical area involvement with different pharmacological sensitivities, as well as the potential for non‐canonical seizure propagation, may both contribute to pharmaco‐resistance (Heinemann *et al*. 1994a). The tonic–clonic pattern of discharges appears to be a feature only of discharges arising in neo‐ and entorhinal cortex, but not hippocampus; when such activity is seen in hippocampus, it appears to be relayed there from the entorhinal cortex. A similar result has been reported previously (Shi *et al*. 2014), which noted that brain slices containing only the dentate and CA territories did not sustain ictal‐like events. In contrast, slices that also included entorhinal cortex and neocortical territories showed ictal‐like events relayed into the hippocampus from the entorhinal cortex. Also, a study of resected human sclerotic hippocampal tissue found that sustained ictal‐like events were almost never recorded in the CA territories (Reyes‐Garcia *et al*. 2018). We have yet to explore the subicular and parasubicular territories.

The hippocampal activity starts very late in the 0 Mg^2+^ model, but very early in the 4‐AP model. To the best of our knowledge, this key difference in these two very widely used models, which illustrates the principle about differing network susceptibility to seizures, has not been reported previously. The hippocampal discharges entrain the other territories in the late 0 Mg^2+^ model, but not in the early 4‐AP activity, illustrating that the entrainment requires an increase in susceptibility (excitability) in the follower territories. Thus, while we emphasize that the explicit explanation of the transition is a shift in the source of the discharges, this must be underpinned by changes at the local network/cellular level, which alter the excitability of the networks.

Another important point, with clinical relevance, is that sudden changes in the local pattern of activity can be indicative of a shift in the source of the pathological driver. Thus, unexplained sudden transitions in discharge patterns may be indicative of multiple foci, an issue of great importance when considering surgical approaches to management. Previously we showed that a change in the direction of propagation of individual discharges is a marker of the passage of the ictal wavefront (Trevelyan *et al*. 2007; Smith *et al*. 2016). Our current study now shows that sudden changes in the pattern of activity recorded in neocortex reflects the appearance, or cessation (suppressed by GABA_B_), of a different pacemaker source, in this case within the hippocampal territories.

The late stage activity, which our studies indicate is a primarily hippocampal pattern, has been termed ‘interictal’ activity by many observers, relating this to the clinical distinction between clinically manifest seizures, which presumably involve some motor territories in the brain, and epileptic electrophysiological discharges that are virtually clinically silent. The implication is that interictal discharges are restricted to areas that are less ‘eloquent’, but that disregards what may be more subtle effects on brain function. Indeed, increasing evidence exists now about the potential impact of interictal discharges on memory (Binnie *et al*. 1987; Kleen *et al*. 2010); such effects, in tandem without an explicit motor component, are entirely consistent with a hippocampal discharge. An important on‐going debate has centred on the clinical significance of these events, specifically with regard to treatments predicated largely on the EEG findings. However, the clear demonstration that these are susceptible to GABA_B_ agonists provides a means to examine this. GABA_B_ agonists have been considered for treating epilepsy previously, but gave mixed results as assessed by seizure control (Terrence *et al*. 1983). We suggest, however, that it might be considered as adjunctive therapy to more conventional anti‐epileptics, with the aim of reducing interictal activity with a presumptive hippocampal origin, and thereby ameliorating memory dysfunction comorbidity. This is also consistent with recent work indicating that focal targeting of dentate function can impact on both memory issues and seizure severity (Liou *et al*. 2018; Scharfman, 2018). Baclofen therapy is not entirely straightforward, because at different doses it appears to induce divergent effects on the hippocampus (Dugladze *et al*. 2013), but it might be possible to calibrate the dose using EEG monitoring in individual patients.

Finally, we provide a proof of principle demonstration of entrainment of epileptiform discharges at a distance, through a non‐synaptic mechanism. This is not mediated through diffusion of [K^+^]_o_, since any rises of [K^+^]_o_ appear to remain very local to the site of neuronal activity. Rather the entrainment is likely to arise through volume conduction of the field potential. Given the size of field fluctuations recorded even outside the skull during seizures, it is reasonable to presume that such entrainment across brain territories might also occur in spontaneous seizures, giving rise to complex patterns of spread. Of course, we stress that this demonstration of a non‐canonical mode of spread does not downgrade the clear importance of conventional, synaptically mediated spread. A notable feature of this pattern of spread is that we only see it in a very particular situation, spreading into tissue that is already hyperexcitable, with a history of repeated epileptiform discharges. Thus, the specific instances of non‐synaptic spread occur only in what we have termed ‘late‐stage’ epileptiform activity, in the 0 Mg^2+^ model; it does not occur with the early hippocampal discharges in 4‐AP, nor in the early neocortical discharges in 0 Mg^2+^. However, the fact that separating the neocortex and hippocampus influences this late stage activity in both directions (the neocortex shows a significant slowing of the rate of discharges, whereas the hippocampal rate increases significantly) indicates that the interactions are indeed bilateral in hyperexcitable networks. This suggests, first, that the neocortical discharges, which tend to last longer than the hippocampal ones, may impose additional refractoriness, and second, that the critical determinant of spread is that the follower network is ‘primed’ for activation. We follow Jefferys's nomenclature (Jefferys, 1995) in avoiding the use of the term ‘ephaptic spread’, since he reserves this term for activation of juxtaposing cells (it derives from the Greek word ‘to touch’), whereas the effect we describe clearly occurs at a distance. We suggest that this occurs through a distant field effect. Even though this might be considered a relatively weak effect, there is, however, an important precedent for this result, whereby epileptiform discharges can be entrained by minimal activation in an already hyperexcitable network: this is the demonstration that bursts of action potentials of a single pyramidal cell can entrain these discharges in disinhibited hippocampal networks (Miles & Wong, 1983). In conclusion, we have demonstrated several key principles of network interactions in epileptic pathophysiology. Although the precise nature in which they will be manifest may be slightly different in a chronically epileptic subject, these phenomena are highly likely to be relevant also *in vivo*, and may inform our interpretation of clinical electrophysiology.

## Additional information

### Competing interests

The authors declare no conflict of interest.

### Author contributions

Experiments and data analysis were performed by N.K.C., R.R.P. and A.J.T. Experiments were conceived by N.K.C., R.R.P. and A.J.T. The manuscript was written by N.K.C. and A.J.T. All authors have read and approved the final version of this manuscript and agree to be accountable for all aspects of the work in ensuring that questions related to the accuracy or integrity of any part of the work are appropriately investigated and resolved. All persons designated as authors qualify for authorship, and all those who qualify for authorship are listed.

### Funding

The work was supported by project grants from Epilepsy Research UK (P1504) and Medical Research Council (UK) (MR/J013250/1 and MR/R005427/1). A.J.T. was the recipient of a Schaefer Scholarship from Columbia University.

## References

[tjp13424-bib-0001] Anastassiou CA , Perin R , Markram H & Koch C (2011). Ephaptic coupling of cortical neurons. Nat Neurosci 14, 217–223. 21240273 10.1038/nn.2727

[tjp13424-bib-0002] Anderson WW , Stasheff SF , Swartzwelder HS & Wilson WA (1990). Regenerative, all‐or‐none electrographic seizures in the rat hippocampal slice in Mg‐free and physiological medium. Brain Res 532, 288–298. 2282522 10.1016/0006-8993(90)91771-8

[tjp13424-bib-0003] Asinof SK , Sukoff Rizzo SJ , Buckley AR , Beyer BJ , Letts VA , Frankel WN & Boumil RM (2015). Independent neuronal origin of seizures and behavioral comorbidities in an animal model of a severe childhood genetic epileptic encephalopathy. PLoS Genet 11, e1005347. 26125563 10.1371/journal.pgen.1005347 PMC4488318

[tjp13424-bib-0004] Avoli M , de Curtis M , Gnatkovsky V , Gotman J , Kohling R , Levesque M , Manseau F , Shiri Z & Williams S (2016). Specific imbalance of excitatory/inhibitory signaling establishes seizure onset pattern in temporal lobe epilepsy. J Neurophysiol 115, 3229–3237. 27075542 10.1152/jn.01128.2015 PMC4946603

[tjp13424-bib-0005] Bernard C , Cossart R , Hirsch JC , Esclapez M & Ben‐Ari Y (2000). What is GABAergic inhibition? How is it modified in epilepsy? Epilepsia 41(Suppl 6), S90–S95. 10999527 10.1111/j.1528-1157.2000.tb01564.x

[tjp13424-bib-0006] Binnie CD , Kasteleijn‐Nolst Trenite DG , Smit AM & Wilkins AJ (1987). Interactions of epileptiform EEG discharges and cognition. Epilepsy Res 1, 239–245. 3504400 10.1016/0920-1211(87)90031-3

[tjp13424-bib-0007] Bragdon AC , Kojima H & Wilson WA (1992). Suppression of interictal bursting in hippocampus unleashes seizures in entorhinal cortex: a proepileptic effect of lowering [K^+^]_o_ and raising [Ca^2+^]_o_ . Brain Res 590, 128–135. 1422827 10.1016/0006-8993(92)91088-v

[tjp13424-bib-0008] Dichter MA & Ayala GF (1987). Cellular mechanisms of epilepsy: a status report. Science 237, 157–164. 3037700 10.1126/science.3037700

[tjp13424-bib-0009] Dreier JP & Heinemann U (1990). Late low magnesium‐induced epileptiform activity in rat entorhinal cortex slices becomes insensitive to the anticonvulsant valproic acid. Neurosci Lett 119, 68–70. 1982961 10.1016/0304-3940(90)90757-z

[tjp13424-bib-0010] Dreier JP & Heinemann U (1991). Regional and time dependent variations of low Mg^2+^ induced epileptiform activity in rat temporal cortex slices. Exp Brain Res 87, 581–596. 1783028 10.1007/BF00227083

[tjp13424-bib-0011] Dreier JP , Zhang CL & Heinemann U (1998). Phenytoin, phenobarbital, and midazolam fail to stop status epilepticus‐like activity induced by low magnesium in rat entorhinal slices, but can prevent its development. Acta Neurol Scand 98, 154–160. 9786610 10.1111/j.1600-0404.1998.tb07286.x

[tjp13424-bib-0012] Dugladze T , Maziashvili N , Borgers C , Gurgenidze S , Haussler U , Winkelmann A , Haas CA , Meier JC , Vida I , Kopell NJ & Gloveli T (2013). GABA_B_ autoreceptor‐mediated cell type‐specific reduction of inhibition in epileptic mice. Proc Natl Acad Sci U S A 110, 15073–15078. 23980149 10.1073/pnas.1313505110 PMC3773756

[tjp13424-bib-0013] Dzhala VI , Kuchibhotla KV , Glykys JC , Kahle KT , Swiercz WB , Feng G , Kuner T , Augustine GJ , Bacskai BJ & Staley KJ (2010). Progressive NKCC1‐dependent neuronal chloride accumulation during neonatal seizures. J Neurosci 30, 11745–11761. 20810895 10.1523/JNEUROSCI.1769-10.2010 PMC3070296

[tjp13424-bib-0014] Ellender TJ , Raimondo JV , Irkle A , Lamsa KP & Akerman CJ (2014). Excitatory effects of parvalbumin‐expressing interneurons maintain hippocampal epileptiform activity via synchronous afterdischarges. J Neurosci 34, 15208–15222. 25392490 10.1523/JNEUROSCI.1747-14.2014 PMC4228130

[tjp13424-bib-0015] Frohlich F & McCormick DA (2010). Endogenous electric fields may guide neocortical network activity. Neuron 67, 129–143. 20624597 10.1016/j.neuron.2010.06.005 PMC3139922

[tjp13424-bib-0016] Fujiwara‐Tsukamoto Y , Isomura Y , Imanishi M , Fukai T & Takada M (2007). Distinct types of ionic modulation of GABA actions in pyramidal cells and interneurons during electrical induction of hippocampal seizure‐like network activity. Eur J Neurosci 25, 2713–2725. 17459104 10.1111/j.1460-9568.2007.05543.x

[tjp13424-bib-0017] Hablitz JJ & Heinemann U (1987). Extracellular K^+^ and Ca^2+^ changes during epileptiform discharges in the immature rat neocortex. Brain Res 433, 299–303. 3690340 10.1016/0165-3806(87)90036-8

[tjp13424-bib-0018] Heinemann U , Draguhn A , Ficker E , Stabel J & Zhang CL (1994a). Strategies for the development of drugs for pharmacoresistant epilepsies. Epilepsia 35(Suppl 5), S10–S21. 10.1111/j.1528-1157.1994.tb05959.x 7518767

[tjp13424-bib-0019] Heinemann U , Dreier J , Leschinger A , Stabel J , Draguhn A & Zhang C (1994b). Effects of anticonvulsant drugs on hippocampal neurons. Hippocampus 4, 291–296. 7842052 10.1002/hipo.450040312

[tjp13424-bib-0020] Heinemann U & Lux HD (1977). Ceiling of stimulus induced rises in extracellular potassium concentration in the cerebral cortex of cat. Brain Res 120, 231–249. 832122 10.1016/0006-8993(77)90903-9

[tjp13424-bib-0021] Huberfeld G , Menendez de la Prida L , Pallud J , Cohen I , Le Van Quyen M , Adam C , Clemenceau S , Baulac M & Miles R (2011). Glutamatergic pre‐ictal discharges emerge at the transition to seizure in human epilepsy. Nat Neurosci 14, 627–634. 21460834 10.1038/nn.2790

[tjp13424-bib-0022] Jefferys JG (1995). Nonsynaptic modulation of neuronal activity in the brain: electric currents and extracellular ions. Physiol Rev 75, 689–723. 7480159 10.1152/physrev.1995.75.4.689

[tjp13424-bib-0023] Jefferys JG & Haas HL (1982). Synchronized bursting of CA1 hippocampal pyramidal cells in the absence of synaptic transmission. Nature 300, 448–450. 6292731 10.1038/300448a0

[tjp13424-bib-0024] Kleen JK , Scott RC , Holmes GL & Lenck‐Santini PP (2010). Hippocampal interictal spikes disrupt cognition in rats. Ann Neurol 67, 250–257. 20225290 10.1002/ana.21896 PMC2926932

[tjp13424-bib-0025] Kramer MA , Truccolo W , Eden UT , Lepage KQ , Hochberg LR , Eskandar EN , Madsen JR , Lee JW , Maheshwari A , Halgren E , Chu CJ & Cash SS (2012). Human seizures self‐terminate across spatial scales via a critical transition. Proc Natl Acad Sci U S A 109, 21116–21121. 23213262 10.1073/pnas.1210047110 PMC3529091

[tjp13424-bib-0026] Lewis DV , Jones LS & Swartzwelder HS (1989). The effects of baclofen and pertussis toxin on epileptiform activity induced in the hippocampal slice by magnesium depletion. Epilepsy Res 4, 109–118. 2792065 10.1016/0920-1211(89)90015-6

[tjp13424-bib-0027] Liou JY , Ma H , Wenzel M , Zhao M , Baird‐Daniel E , Smith EH , Daniel A , Emerson R , Yuste R , Schwartz TH & Schevon CA (2018). Role of inhibitory control in modulating focal seizure spread. Brain 141, 2083–2097. 29757347 10.1093/brain/awy116 PMC6022627

[tjp13424-bib-0028] Martinet LE , Fiddyment G , Madsen JR , Eskandar EN , Truccolo W , Eden UT , Cash SS & Kramer MA (2017). Human seizures couple across spatial scales through travelling wave dynamics. Nat Commun 8, 14896. 28374740 10.1038/ncomms14896 PMC5382286

[tjp13424-bib-0029] McCormick DA & Contreras D (2001). On the cellular and network bases of epileptic seizures. Annu Rev Physiol 63, 815–846. 11181977 10.1146/annurev.physiol.63.1.815

[tjp13424-bib-0030] Miles R & Wong RK (1983). Single neurones can initiate synchronized population discharge in the hippocampus. Nature 306, 371–373. 6316152 10.1038/306371a0

[tjp13424-bib-0031] Miles R , Wong RK & Traub RD (1984). Synchronized afterdischarges in the hippocampus: contribution of local synaptic interactions. Neuroscience 12, 1179–1189. 6090986 10.1016/0306-4522(84)90012-5

[tjp13424-bib-0032] Mody I , Lambert JD & Heinemann U (1987). Low extracellular magnesium induces epileptiform activity and spreading depression in rat hippocampal slices. J Neurophysiol 57, 869–888. 3031235 10.1152/jn.1987.57.3.869

[tjp13424-bib-0033] Moody WJ , Futamachi KJ & Prince DA (1974). Extracellular potassium activity during epileptogenesis. Exp Neurol 42, 248–263. 4824976 10.1016/0014-4886(74)90023-5

[tjp13424-bib-0034] Morrisett RA , Lewis DV , Swartzwelder HS & Wilson WA (1993). Antiepileptic effects of GABA_b_ receptor activation in area CA3 of rat hippocampus. Brain Res 600, 235–242. 8382098 10.1016/0006-8993(93)91378-6

[tjp13424-bib-0035] Pallud J , Le Van Quyen M , Bielle F , Pellegrino C , Varlet P , Labussiere M , Cresto N , Dieme MJ , Baulac M , Duyckaerts C , Kourdougli N , Chazal G , Devaux B , Rivera C , Miles R , Capelle L & Huberfeld G (2014). Cortical GABAergic excitation contributes to epileptic activities around human glioma. Sci Transl Med 6, 244ra289. 10.1126/scitranslmed.3008065 PMC4409113 25009229

[tjp13424-bib-0036] Parrish RR & Trevelyan AJ (2018). Stress‐testing the brain to understand its breaking points. J Physiol 596, 2033–2034. 29604061 10.1113/JP276184 PMC5983151

[tjp13424-bib-0037] Pavlov I , Kaila K , Kullmann DM & Miles R (2013). Cortical inhibition, pH and cell excitability in epilepsy: what are optimal targets for antiepileptic interventions? J Physiol 591, 765–774. 22890709 10.1113/jphysiol.2012.237958 PMC3591695

[tjp13424-bib-0038] Prince DA & Connors BW (1984). Mechanisms of epileptogenesis in cortical structures. Ann Neurol 16(Suppl), S59–S64. 6095743 10.1002/ana.410160710

[tjp13424-bib-0039] Reyes‐Garcia SZ , Scorza CA , Araujo NS , Ortiz‐Villatoro NN , Jardim AP , Centeno R , Yacubian EMT , Faber J & Cavalheiro EA (2018). Different patterns of epileptiform‐like activity are generated in the sclerotic hippocampus from patients with drug‐resistant temporal lobe epilepsy. Sci Rep 8, 7116. 29740014 10.1038/s41598-018-25378-9 PMC5940759

[tjp13424-bib-0040] Scharfman HE (2018). Controlling learning and epilepsy together. Science 359, 740–741. 29449476 10.1126/science.aas8993 PMC6044721

[tjp13424-bib-0041] Schevon CA , Weiss SA , McKhann G Jr , Goodman RR , Yuste R , Emerson RG & Trevelyan AJ (2012). Evidence of an inhibitory restraint of seizure activity in humans. Nat Commun 3, 1060. 22968706 10.1038/ncomms2056 PMC3658011

[tjp13424-bib-0042] Shi YJ , Gong XW , Gong HQ , Liang PJ , Zhang PM & Lu QC (2014). Effect of the entorhinal cortex on ictal discharges in low‐Mg^2+^‐induced epileptic hippocampal slice models. Neural Plast 2014, 205912. 24729906 10.1155/2014/205912 PMC3960561

[tjp13424-bib-0043] Smith EH , Liou JY , Davis TS , Merricks EM , Kellis SS , Weiss SA , Greger B , House PA , McKhann Ii GM , Goodman RR , Emerson RG , Bateman LM , Trevelyan AJ & Schevon CA (2016). The ictal wavefront is the spatiotemporal source of discharges during spontaneous human seizures. Nat Commun 7, 11098. 27020798 10.1038/ncomms11098 PMC4820627

[tjp13424-bib-0044] Somjen GG & Giacchino JL (1985). Potassium and calcium concentrations in interstitial fluid of hippocampal formation during paroxysmal responses. J Neurophysiol 53, 1098–1108. 3998794 10.1152/jn.1985.53.4.1098

[tjp13424-bib-0045] Swartzwelder HS , Bragdon AC , Sutch CP , Ault B & Wilson WA (1986a). Baclofen suppresses hippocampal epileptiform activity at low concentrations without suppressing synaptic transmission. J Pharmacol Exp Ther 237, 881–887. 3012073

[tjp13424-bib-0046] Swartzwelder HS , Lewis DV , Anderson WW & Wilson WA (1987). Seizure‐like events in brain slices: suppression by interictal activity. Brain Res 410, 362–366. 3594246 10.1016/0006-8993(87)90339-8

[tjp13424-bib-0047] Swartzwelder HS , Sutch CP & Wilson WA (1986b). Attenuation of epileptiform bursting by baclofen: reduced potency in elevated potassium. Exp Neurol 94, 726–734. 3780917 10.1016/0014-4886(86)90250-5

[tjp13424-bib-0048] Terrence CF , Fromm GH & Roussan MS (1983). Baclofen. Its effect on seizure frequency. Arch Neurol 40, 28–29. 6848083 10.1001/archneur.1983.04050010048011

[tjp13424-bib-0049] Traub RD & Wong RK (1982). Cellular mechanism of neuronal synchronization in epilepsy. Science 216, 745–747. 7079735 10.1126/science.7079735

[tjp13424-bib-0050] Trevelyan AJ , Baldeweg T , van Drongelen W , Yuste R & Whittington M (2007). The source of afterdischarge activity in neocortical tonic‐clonic epilepsy. J Neurosci 27, 13513–13519. 18057209 10.1523/JNEUROSCI.3005-07.2007 PMC6673106

[tjp13424-bib-0051] Trevelyan AJ & Schevon CA (2013). How inhibition influences seizure propagation. Neuropharmacology 69, 45–54. 22722026 10.1016/j.neuropharm.2012.06.015

[tjp13424-bib-0052] Weiss SA , Banks GP , McKhann GM Jr , Goodman RR , Emerson RG , Trevelyan AJ & Schevon CA (2013). Ictal high frequency oscillations distinguish two types of seizure territories in humans. Brain 136, 3796–3808. 24176977 10.1093/brain/awt276 PMC3859220

[tjp13424-bib-0053] Whittington MA , Traub RD & Jefferys JG (1995). Erosion of inhibition contributes to the progression of low magnesium bursts in rat hippocampal slices. J Physiol 486, 723–734. 7473233 10.1113/jphysiol.1995.sp020848 PMC1156560

[tjp13424-bib-0054] Yu FH , Mantegazza M , Westenbroek RE , Robbins CA , Kalume F , Burton KA , Spain WJ , McKnight GS , Scheuer T & Catterall WA (2006). Reduced sodium current in GABAergic interneurons in a mouse model of severe myoclonic epilepsy in infancy. Nat Neurosci 9, 1142–1149. 16921370 10.1038/nn1754

[tjp13424-bib-0055] Zhang CL , Dreier JP & Heinemann U (1995). Paroxysmal epileptiform discharges in temporal lobe slices after prolonged exposure to low magnesium are resistant to clinically used anticonvulsants. Epilepsy Res 20, 105–111. 7750506 10.1016/0920-1211(94)00067-7

[tjp13424-bib-0056] Ziburkus J , Cressman JR , Barreto E & Schiff SJ (2006). Interneuron and pyramidal cell interplay during in vitro seizure‐like events. J Neurophysiol 95, 3948–3954. 16554499 10.1152/jn.01378.2005 PMC1469233

